# DHCR7 inhibition ameliorates MetALD and HCC in mice and human 3D liver spheroids

**DOI:** 10.1016/j.jhepr.2025.101415

**Published:** 2025-04-05

**Authors:** Gen Yamamoto, Raquel Carvalho-Gontijo Weber, Wonseok Lee, Vivian Zhang, Haeum Jang, Sadatsugu Sakane, Xiao Liu, Hyun Young Kim, David A. Brenner, Na Li, Tatiana Kisseleva

**Affiliations:** 1 Department of Medicine, University of California, San Diego, La Jolla, CA, USA; 2 Department of Surgery, University of California, San Diego, La Jolla, CA, USA; 3 Department of Surgery, Kyoto University Graduate School of Medicine, Kyoto, Japan; 4 College of Pharmacy, Gachon University, Incheon, Republic of Korea; 5 College of Pharmacy, Dankook University, Cheonan, Chungnam, Republic of Korea; 6 Sanford Burnham Prebys Medical Discovery Institute, La Jolla, CA, USA; 7 College of Medical Technology, Shanghai University of Medicine and Health Sciences, Shanghai, P. R. China

**Keywords:** Alcohol-induced liver injury, Cholesterol synthesis, Steatosis, Inflammation, Fibrosis, Tumor growth

## Abstract

**Background & Aims:**

Metabolic dysfunction and alcohol-associated liver disease (MetALD) results in the development of liver steatosis, inflammation, fibrosis, and hepatocellular carcinoma (HCC). *De novo* lipogenesis and cholesterol synthesis play an important role in the pathogenesis of MetALD. DHCR7 (7-dehydrocholesterol reductase) regulates the last stages of cholesterol production.

**Methods:**

We investigated whether targeting DHCR7 can ameliorate the development of MetALD and HCC using experimental models and 3D human liver spheroids.

**Results:**

Here, we demonstrate that partial genetic ablation of the *Dhcr7* gene and pharmacological blockade of DHCR7 activity with the AY9944 inhibitor suppresses hepatic steatosis (↓ lipid area, n = 15; *p* <0.001), inflammation (↓ F4/80, n = 6; *p* <0.01), fibrosis (↓ Sirius red, n = 6; *p* <0.01), and HCC (↓ AFP/YAP, n = 6; *p* <0.01) in diethylnitrosamine (DEN)-challenged high-fat diet (HFD) + ethanol (EtOH)-fed mice treated with AY9944 compared with control mice. To translate our findings, the effect of DHCR7 was tested using 3D human liver spheroids, which mimicked MetALD and MetALD-HCC. MetALD liver spheroids were composed of primary human hepatocytes, non-parenchymal cells, and hepatic stellate cells. In contrast, in MetALD-HCC spheroids, the HCC cell line HepG2 was used instead of hepatocytes. Therapeutic administration of AY9944 inhibited inflammation (↓ *TNF*, *p* <0.05) and fibrosis in MetALD spheroids (↓ *ACTA2*, *p* <0.001; *COL1A1*, *p* <0.05; *TIMP1*, *p* <0.01; *SERPINE1*, *p* <0.05). In turn, dsiRNA-based knockdown of DHCR7 reduced HepG2 proliferation (↓ *PCNA*, *p* <0.05; *CCNE*, *p* <0.05) and expression of MetALD-HCC markers (↓ *AFP*, *p* <0.05; *GPC3*, *p* <0.05; *YAP*, *p* <0.01).

**Conclusions:**

Our data demonstrate that targeting DHCR7 can become a strategy for the treatment of MetALD and HCC.

**Impact and implications:**

This study demonstrates the critical role of *de novo* lipogenesis and cholesterol synthesis in the pathogenesis of metabolic dysfunction and alcohol-associated liver disease (MetALD) and its progression to hepatocellular carcinoma (HCC). Our findings identified that the upregulation of DHCR7 contributes to the pathogenesis of MetALD and its inhibition suppresses hepatic steatosis, inflammation, fibrosis, and tumor proliferation. These findings are significant for researchers and clinicians, as they establish that genetic and pharmacological inhibition of DHCR7 is effective in both experimental models and translational 3D human liver spheroids. The results uncover the translational potential of DHCR7-targeted therapies for MetALD and HCC, offering practical implications for the development of novel treatment strategies. Further studies are necessary to optimize these approaches and address potential methodological limitations.

## Highlights

• DHCR7, a key enzyme in cholesterol production, plays a pivotal role in the pathogenesis of experimental MetALD.
• Partial genetic ablation or pharmacological inhibition of DHCR7 reduced steatosis, inflammation, fibrosis, and HCC development in mice.
• Treatment with AY9944 effectively inhibited steatosis, inflammation, and fibrosis in the MetALD 3D human liver spheroids.
• *DHCR7* knockdown effectively inhibited HepG2 proliferation in the MetALD-HCC 3D human liver spheroids.
• DHCR7 can become a promising therapeutic target to treat MetALD and prevent HCC progression.

## Graphical abstract

**Figure:**
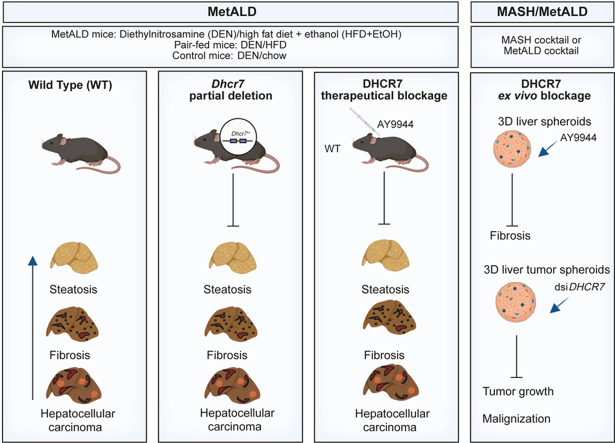

## Introduction

Toxic chronic liver injury, caused by HBV/HCV infections, metabolic dysfunction-associated steatohepatitis (MASH), and metabolic dysfunction and alcohol-associated liver disease (MetALD), often results in the development of liver fibrosis and hepatocellular carcinoma (HCC).^1^ Consistent with the global epidemic of MASH, alcohol-induced liver injury often occurs in obese patients,^2^ leading to the development of MetALD. With the introduction of new therapies, the incidence of HBV/HCV has declined, whereas the incidence of MASH and MetALD, both of which typically progress from hepatic steatosis to steatohepatitis, fibrosis, cirrhosis, and HCC,^1^ are rapidly rising. Alcohol-associated metabolic injury causes apoptosis of hepatocytes, recruitment of inflammatory cells into the damaged liver, secretion of inflammatory and fibrogenic cytokines (*e.g.* IL-6, TNF, IL-1β, and TGFβ1), and activation of collagen type I-producing hepatic stellate cells (HSCs)/myofibroblasts, which make the liver fibrotic.^1^ Here, we investigated the role of *de novo* lipogenesis in the pathogenesis of MetALD and HCC using experimental models of MetALD in mice and human 3D liver spheroids. Specifically, the role of cholesterol synthesis in MetALD and HCC was assessed.

Alcohol is metabolized by hepatocytes, which produce acetaldehyde and acetate and increase fatty acid and cholesterol synthesis.^2^ This results in inflammation and fibrosis.^3^ Hepatocytes respond to metabolic injury by lipid accumulation (mainly triglycerides and phospholipids), which is regulated on several levels: *de novo* lipogenesis, lipid secretion (VLDL), and inhibition of β-oxidation.^4^^,^^5^ Excessive *de novo* lipogenesis is instigated by oxidative stress, TNF/TNFR1, and IL-17A/IL17RA signaling,^6^^,^^7^ which trigger Caspase2-S1P/S2P pathway-dependent activation of element-binding proteins 1 and 2 (SREBP1 and SREBP2),^8^ transcription factors that control the production of lipogenic genes and enzymes, such as 7-dehydrocholesterol reductase (DHCR7) and DHCR24, which catalyze the final steps of cholesterol and fatty acid synthesis.^9^ DHCR7 is the sole enzyme that regulates the final steps of cholesterol production^10^ and therefore may serve as a primary target for the treatment of MetALD and HCC. Excessive accumulation of cholesterol produces a cytotoxic effect on hepatocytes, triggering their de-differentiation into premalignant progenitors.

Despite extensive studies, the functions of the DHCR7 enzyme in MetALD are not well understood. In support, downregulation of cholesterol synthesis is linked to tumor suppression.^11^^,^^12^ *Dhcr7*^*–/–*^ mice^13^^,^^14^ develop symptoms of the autosomal recessive developmental disorder Smith–Lemli–Opitz syndrome,^15^ leading to suppression of cholesterol synthesis and accumulation of 7-dehydrocholesterol (7-DHC). In the absence of DHCR7, 7-DHC is converted into vitamin D_3_,^16^^,^^17^ which possesses strong anti-proliferative and anti-inflammatory properties.

Here we evaluated the role of DHCR7 in the pathogenesis of MetALD and HCC in alcohol-injured wild-type (WT) and *Dhcr7*^*+/–*^ mice and determined that suppression of DHCR7 ameliorates hepatic steatosis, inflammation, and fibrosis and reduces the incidences of alcohol-induced HCC. Moreover, therapeutic administration of DHCR7 inhibitor AY9944 attenuates MetALD and HCC in WT mice. To translate our findings in mice, a model of MetALD and MetALD-HCC “in a dish” was generated using 3D human liver spheroids. The role of DHCR7 in tumors and the tumor microenvironment has been dissected. Overall, our data demonstrate that suppression of DHCR7-dependent cholesterol synthesis can be used for the treatment of MetALD and HCC.

## Materials and methods

### Mice

*Dhcr7*^*+/–*^ mice (Jackson Laboratory, Bar Harbor, ME, USA) and WT littermates (C57BL/6, males) were maintained under specific pathogen-free conditions at UCSD according to IACUC protocol S07088.

### Experimental model of MetALD and HCC

DHCR7^+/–^ and WT mice (C57BL/6, 14 days old) were injected i.p. with diethylnitrosamine (DEN) (25 mg/kg body weight; Sigma). DEN-challenged mice (3 months old) were subjected to isocaloric liquid ethanol (EtOH) feeding (HFD + EtOH: Lieber DeCarli ethanol diet #710362, Dyets Inc.), pair feeding (calorie-matched HFD: Lieber DeCarli high-fat diet #710142, Dyets Inc.), or chow feeding (diet #5010, LAB) for 18 weeks. The concentration of alcohol was gradually increased from 1% to 2% and 3% (*v*/*v*, throughout 3 weeks) and maintained at 3.5% (*v*/*v*) in EtOH-fed mice for the rest of the feeding (total 18 weeks).^9^

### Analysis of human livers

RNA sequencing (RNA-seq) data from 50 normal liver tissues and 371 primary liver HCC samples (project TCGA-LIHC) were analyzed using FPKM (fragments per kilobase of transcript per million mapped reads) to normalize expression levels in RNA-seq, and were aligned to the clinical metadata for each patient. Statistical significance between DHCR7 expression in HCC and non-tumor tissues was assessed using Student’s *t* test in R (version 3.4.2). Overall survival (OS) analysis was conducted using a web tool (http://kmplot.com/analysis).^18^ This web tool integrates transcriptomic data and OS information from patients with HCC. Clinically relevant patient characteristics were also documented within the database. Multivariate analyses were conducted to examine the association between OS and disease stage or hepatitis virus infection, in conjunction with DHCR7 expression. OS curves were generated to illustrate differences in survival outcomes, which were statistically evaluated using the log-rank test based on existing data sources and clinical records (Table S1). A *p* value of <0.05 was considered statistically significant. Human HCC slides were purchased from Aifang Biological. Immunohistochemistry was performed using anti-human DHCR7 Ab (D122232, Sangon Biotech; 1:200 dilution).

### Tumor evaluation

AFP^+^YAP^+^ HCC (≈3 mm) and non-tumor tissues were microdissected from HFD + EtOH-, pair-, and chow-fed *Dhcr7*^*+/–*^ mice and littermates and were used for quantitative real-time PCR (qRT-PCR). HCC tumor burden^19^ was calculated as tumor volume ($x^{2}.\frac{y}{2}$ mm^3^): body weight (g) × 100%, where x denotes the smallest tumor diameter and y is the largest tumor diameter. Data are presented as percentages. Tumors were harvested (*n* >3 per mouse) and analyzed.

### Immunohistochemistry

Livers were formalin-fixed and stained with H&E, Sirius red, or anti-αSMA (Abcam), anti-Desmin (Thermo Scientific), anti-F4/80 (eBioscience), anti-AFP (Biocare Medical), anti-YAP (Cell Signaling), and anti-phospho-STAT3 Ab (Cell Signaling), followed by DAB staining (Vector). Images were taken using an Olympus (Tokyo, Japan) microscope and analyzed by a pathologist in a double-blinded manner. The positive area was calculated as a percentage using ImageJ.^9^

### qRT-PCR

Total RNA was isolated using the Purelink RNA Mini Kit (Life Technologies). qRT-PCR was performed on a QuantStudio 3 (Life Technologies), and expression levels were normalized to HPRT using the ΔΔ CT method (Invitrogen).

### Analysis of liver function

Serum alanine aminotransferase (ALT) was measured using Infinity™ ALT (Thermo Scientific). Serum and hepatic levels of triglycerides, cholesterol, fatty acids, and bile acids were measured using the Triglyceride Reagent Set (Pointe Scientific), Total Cholesterol Assay Kit (Cell Biolabs), Free Fatty Acids, Half-micro Test (Roche), and Mouse Total Bile Acids Kit (Crystal Chem).

### Vitamin D_3_

Serum levels of vitamin D_3_ were measured using ELISA (Aviva Systems Biology).^9^

### HCC cell line and reagents

Mouse green fluorescent protein (GFP)-labeled HCC cell line Hepa 1-6 and human HCC cell line HepG2 (ATCC, Manassas, VA, USA) were cultured in DMEM (Gibco-BRL) supplemented with 10% FBS (Gemini-Bio) and 1% penicillin–streptomycin (Gibco). AY9944 (in PBS; Cayman) was used for the inhibition of DHCR7 activity *in vitro*.

### Adoptive transfer of Hepa 1-6 into livers of recipient WT mice

WT mice were fed with HFD + EtOH for 2 weeks. Hepa 1-6 cells (1 × 10^6^ cells in PBS) were injected into the spleens of WT mice, followed by immediate splenectomy.^20^ Mice continued to be fed HFD + EtOH and were euthanized 2 weeks later. The total number of visible surface tumors or GFP-positive tumors per liver was calculated.^21^ Gross liver lobes (caudate and left lobes) were imaged using a fluorescence microscope (10× magnification). The mean fluorescence intensity (MFI) of GFP per lobe per liver was calculated for each image using ImageJ. The results are presented as the average MFI from eight images per mouse.

### Generation of MASH and MetALD human liver spheroids

MASH human liver spheroids were generated using human liver cells containing isolated steatotic hepatocytes (3 × 10^5^) + non-parenchymal cells (NPCs; 1.5 × 10^5^) + HSCs (0.8 × 10^5^), cultured in 96-well Ultra-low Attachment Plates (Corning, 454552) with a MASH cocktail (160 μM palmitate, 160 μM oleate, 10 mM fructose, 5.5 mM glucose, 10 μg/ml LPS, and 1 ng/ml TGFβ1) for 9 days. MetALD human liver spheroids were generated using the same cell composition and were cultured in a MetALD cocktail (MASH cocktail supplemented with 100 mM ethanol) for an additional 9 days. A total of 100 μl of culture medium per well was replaced with an equal volume of fresh MASH cocktail or MetALD cocktail every 2 days.^24^ Spheroids were harvested and analyzed by immunohistochemistry, qRT-PCR (16 spheroids per sample), and Western blotting (48 spheroids per sample).

### Generation of MetALD-HCC human liver spheroids

The human HCC cell line HepG2 (5 × 10^4^) was transfected with DHCR7-targeting or scrambled (control) dsiRNA (Integrated DNA Technologies) for 48 h (using 10 nM Lipofectamine RNAiMAX, Invitrogen, 13778075). DHCR7 mRNA expression was analyzed using qRT-PCR. More than three hairpins per gene were tested, and one hairpin with the highest knockdown efficiency was selected. DHCR7-knockdown HepG2 cells (3 × 10^5^) were used to generate human liver spheroids containing NPCs (1.5 × 10^5^) and HSCs (0.8 × 10^5^) and were cultured in a MetALD cocktail for 7 days.^21^ The volume of spheroids was calculated using the following formula: (L × I^2^) × π/6, where L and I denote the long and short diameters of the spheroids, respectively.

### Western blot analysis

Spheroids were harvested and lysed in RIPA buffer containing a protease and phosphatase inhibitor cocktail (Sigma). The proteins were separated by SDS-PAGE and transferred to polyvinylidene difluoride membranes. The membranes were blocked in 5% non-fat milk and incubated with the indicated primary antibodies. After incubation with secondary antibodies, proteins were detected using SuperSignal West Pico Plus chemiluminescence substrate (Thermo Scientific). The primary antibodies and dilutions were as follows: β-actin (A5441, Sigma-Aldrich, 1:5,000), collagen type I (72026S, Cell Signaling Technology, 1:1,000), CYP2E1 (AB1252, Millipore, 1:1,000), Perilipin 2 (NB110-40877, Novus Biologicals, 1:1,000), PAI-1 (13801-1-AP, Proteintech, 1:1,000), ɑSMA (ab5694, Abcam, 1:2,000), and AFP (4448S, Cell Signaling Technology, 1:1,000).

### Statistical analysis

All data are shown as mean ± SD. Comparisons between the two groups were analyzed using the unpaired two-tailed Student’s *t* test. Comparisons involving three or more groups were analyzed using ANOVA. ANOVA with Dunnett’s test was used for comparing multiple mouse groups or treatments with a control. ANOVA with a Bonferroni test was used for multiple pairwise comparisons between different groups. A *p* value of <0.05 was considered statistically significant. The analyses were performed using GraphPad Prism software (GraphPad, San Diego, CA, USA).

## Results

### DEN-challenged HFD + EtOH-fed WT mice developed MetALD with HCC

**Fig. 1:**
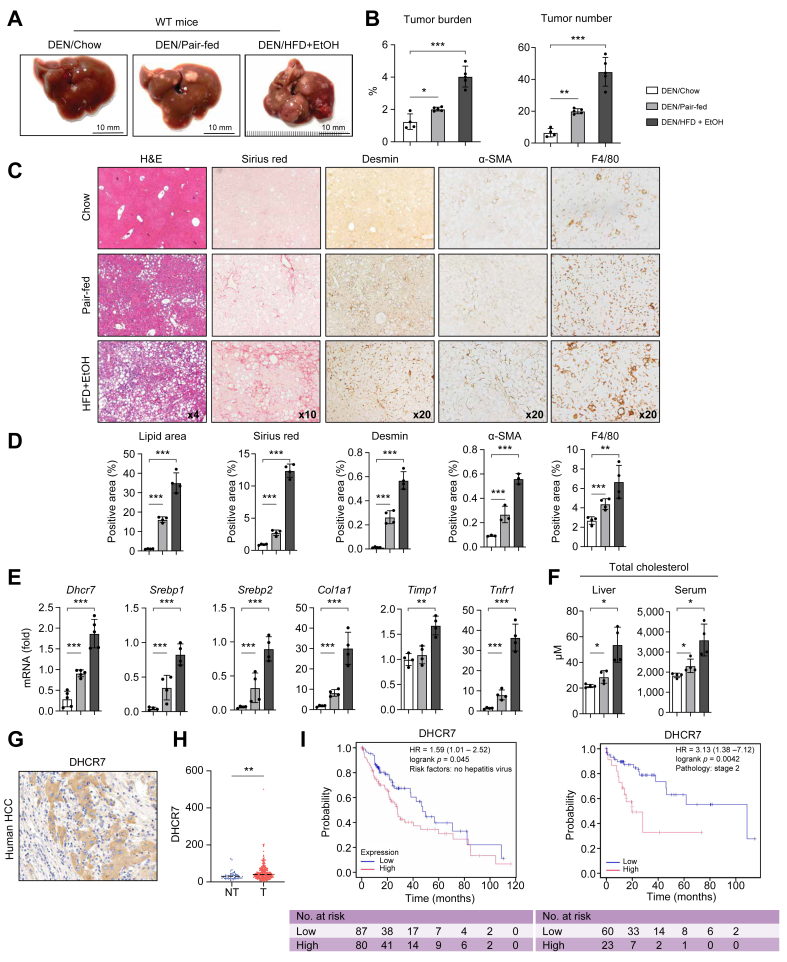
Alcohol accelerates the development of liver injury and HCC in DEN-challenged HFD-fed WT mice. (A) Gross liver images from DEN/chow-, DEN/pair-, and DEN/HFD + EtOH-fed WT mice (n = 3–5 per group). (B) Tumor burden and tumor numbers were calculated. (C, D) Livers were stained with H&E and Sirius red or immunostained for Desmin, αSMA, and F4/80. Area of positive staining was calculated as a percentage. Representative micrographs. Images were taken using 10 × objectives. (E) Expression of selected genes was evaluated using qRT-PCR. (F) Levels of serum and hepatic cholesterol were measured. Statistical analysis was performed using two-tailed Student’s *t* test. Data are mean ± SD; ∗*p* <0.05, ∗∗*p* <0.01, ∗∗∗*p* <0.001. (G, H) Expression of *DHCR7* mRNA was analyzed in human HCC *vs*. non-tumor tissues using two methods: (G) immunohistochemistry (representative image is shown) and (H) RNA-seq analysis of DHCR7 (Student’s *t* test was performed for statistical evaluation). (I) OS in patients was analyzed using the Kaplan–Meier Plotter web server. The left panel represents patients without hepatitis virus infection, and the right panel includes patients with stage 2 HCC. Patients with high DHCR7 expression are shown in red, whereas those with low expression are represented by the blue line. The log-rank *p* value indicates the overall significance of the analysis, and HR represents the hazard ratio. The number of patients at each survival time point is listed below the curve. DEN, diethylnitrosamine; DHCR7, 7-dehydrocholesterol reductase; EtOH, ethanol; HCC, hepatocellular carcinoma; HFD, high-fat diet; OS, overall survival; qRT-PCR, quantitative real-time PCR; RNA-seq, RNA sequencing; WT, wild-type.

WT male mice (C57BL/6, 14 days old, n = 5–6 per group) were injected with a single dose of DEN (25 mg/kg, i.p.). Starting at 3 months of age, the mice were fed a liquid diet containing either HFD + EtOH (EtOH-fed mice) or calorie-matched HFD (pair-fed mice). HCC was assessed in all groups of mice after 18 weeks of feeding and compared with that observed in DEN-WT mice fed normal chow (chow-fed mice) for 9 months (as no tumors were detected in chow-fed mice after 18 weeks). Alcohol strongly accelerated tumorigenesis in DEN-challenged HFD + EtOH (DEN/HFD + EtOH)-fed WT mice (*vs*. DEN/pair-fed mice; Fig. 1A and B). DEN/HFD + EtOH-fed WT mice developed more tumors than DEN-challenged pair-fed or chow-fed WT mice, as shown by the increased number of visible tumors in the gross liver images of these mice (Fig. 1A) and high tumor burden (≈**↑**2-fold *vs*. DEN/pair-fed mice; Fig. 1B). Consistently, the average diameter of liver tumors was greater in DEN/HFD + EtOH-fed WT mice (*vs*. DEN/pair-fed WT mice). Liver histopathology demonstrated that DEN/HFD + EtOH-fed WT mice developed severe steatosis (*vs*. DEN/pair-fed mice or DEN/chow-fed WT mice, which showed no steatosis; Fig. 1C and D). Moreover, liver injury, inflammation, and fibrosis were increased in DEN/HFD + EtOH-fed WT mice compared with pair-fed mice, as shown by increased lipid area and positive staining for Sirius red (↑4-fold) and F4/80 (↑1.5-fold). In addition, mRNA expression of *Col1a1* and *Timp1* was increased in alcohol-fed mice compared with the pair-fed mice (Fig. 1E). The development of MetALD in WT mice was associated with increased hepatic and serum levels of cholesterol and increased liver mRNA expression of *Srebp1*, *Srebp2*, and *Dhcr7* (Fig. 1E and F), suggesting that chronic alcohol exposure activates *de novo* lipogenesis and cholesterol synthesis, significantly worsening liver pathology.

### Increased expression of DHCR7 in human HCC is associated with high mortality in patients

Expression of DHCR7 was evaluated by immunohistochemistry in patients with HCC (n = 3). Increased immunoreactivity for DHCR7 was detected in human HCC compared with non-tumor liver tissues (Fig. 1G). Furthermore, RNA-seq-based DHCR7 gene expression was analyzed in 50 normal liver tissues and 371 primary liver HCC samples from the TCGA database (project TCGA-LIHC) and aligned with the clinical metadata for each patient. The expression levels of *DHCR7* mRNA were significantly elevated in liver tumors compared with the non-tumor liver tissues (Fig. 1H). To assess the clinical relevance of our findings, patients with HCC were evaluated for OS using a Kaplan–Meier Plotter web tool (http://kmplot.com/analysis). The results indicated that among patients with HCC without hepatitis virus infection, high DHCR7 expression was significantly associated with poorer survival probability compared with those with low DHCR7 expression (*p* <0.05). A similar trend was observed in patients with stage 2 HCC. These findings suggest that DHCR7 expression levels are associated with clinical characteristics and may influence patient survival.

### DEN/HFD + EtOH-fed *Dhcr7*^*+/–*^ mice developed fewer tumors compared with WT mice

**Fig. 2:**
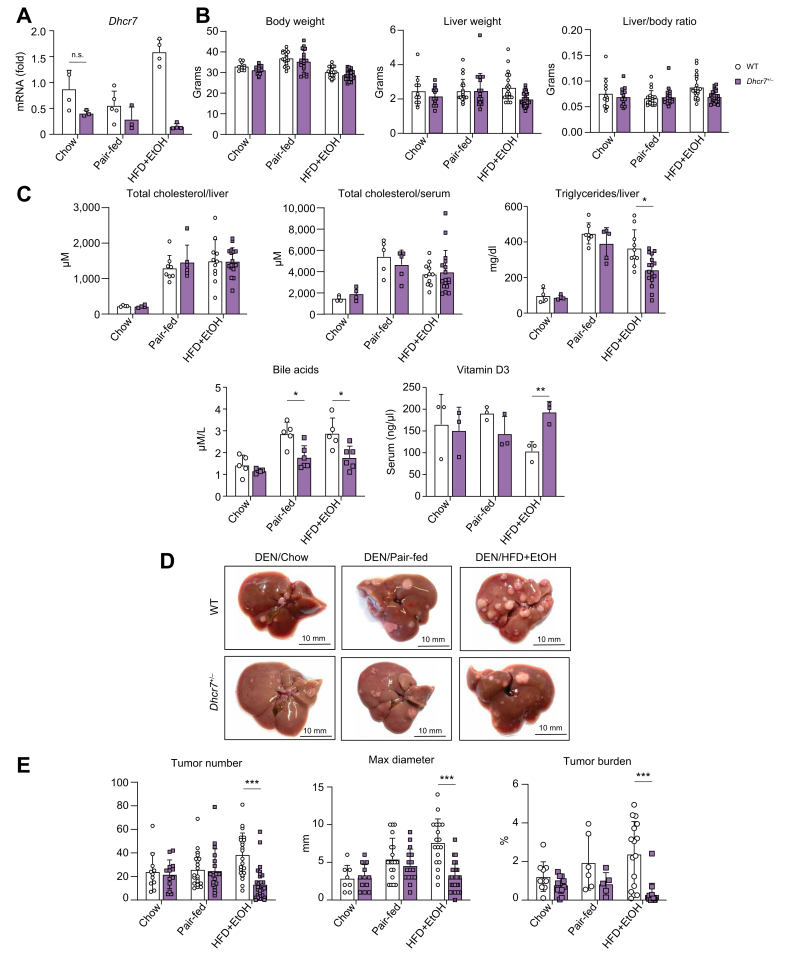
DEN/HFD + EtOH-fed *Dhcr7*^*+/–*^ mice developed fewer tumors than DEN/HFD + EtOH-fed WT littermates. (A) Expression of DHCR7 mRNA in DEN/chow-, DEN/pair-, and DEN/HFD + EtOH-fed WT and *Dhcr7*^*+/–*^ mice (n ≤10 per group). (B) Body weight, liver weight, and liver-to-body weight ratio were calculated. (C) Levels of total serum and hepatic cholesterol, serum triglycerides, bile acids, and vitamin D_3_ were measured. (D) Gross liver images of WT and *Dhcr7*^*+/–*^ mice. (E) Tumor number, diameter, and burden were calculated. Livers were stained with H&E, and positive area was calculated as a percentage (10× objectives). Data are mean ± SD; ∗*p* <0.05, ∗∗*p* <0.01, ∗∗∗*p* <0.001, two-tailed Student’s *t* test. DEN, diethylnitrosamine; DHCR7, 7-dehydrocholesterol reductase; EtOH, ethanol; HFD, high-fat diet; WT, wild-type.

The role of DHCR7 in the pathogenesis of MetALD was examined in the age-matched heterozygous *Dhcr7*^*+/–*^ and WT male littermates (C57BL/6, n = 10–12 per group). *Dhcr7*^*+/–*^ and WT male mice (14 days old) were challenged with DEN and then fed with HFD + EtOH- or pair-fed for 18 weeks or fed chow for 9 months. As expected, reducing the copy number of the *Dhcr7* gene by 50% reduced expression of *Dhcr7* mRNA in *Dhcr7*^*+/–*^ mice (*vs*. WT mice; Fig. 2A). We hypothesized that partial deletion of the *Dhcr7* gene would inhibit alcohol-induced steatosis, inflammation, fibrosis, and HCC in DEN/HFD + EtOH-fed *Dhcr7*^*+/–*^ mice *vs*. WT mice treated under the same conditions.

The body weight was equally increased in both DEN/HFD + EtOH-fed WT and *Dhcr7*^*+/–*^ mice compared with pair-fed WT mice or chow-fed mice. The liver-to-body weight ratio was not significantly different between HFD + EtOH-fed WT and *Dhcr7*^*+/–*^ mice, although *Dhcr7*^*+/–*^ mice tended to have a lower liver-to-body weight ratio than WT mice (Fig. 2B). Consistent with previous reports,^14^ no significant differences in hepatic or serum cholesterol levels were detected in HFD + EtOH-fed WT and D*hcr7*^+/–^ mice (Fig. 2C). In contrast, hepatic triglycerides and bile acids were reduced in HFD + EtOH-fed *Dhcr7*^*+/–*^ mice. The levels of vitamin D_3_ were increased, consistent with a shift in the conversion of DHCR7 substrate, 7-dehydrocholesterol (7-DHC), toward vitamin D_3_^22^ (Fig. 2C). Despite similar cholesterol levels, HFD + EtOH-fed *Dhcr7*^+/–^ mice were protected from tumorigenesis, steatosis, fibrosis, and inflammation. Macroscopically, very few tumors were detected in DEN/ HFD + EtOH-fed *Dhcr7*^*+/–*^ mice (Fig. 2D). DEN/HFD + EtOH-fed *Dhcr7*^*+/–*^ mice developed fewer (↓2-fold) and smaller tumors than WT mice, as shown by reduced (↓3-fold) tumor burden (Fig. 2E).

### Development of HCC was suppressed in DEN/HFD + EtOH-fed *Dhcr7*^*+/–*^ mice

**Fig. 3:**
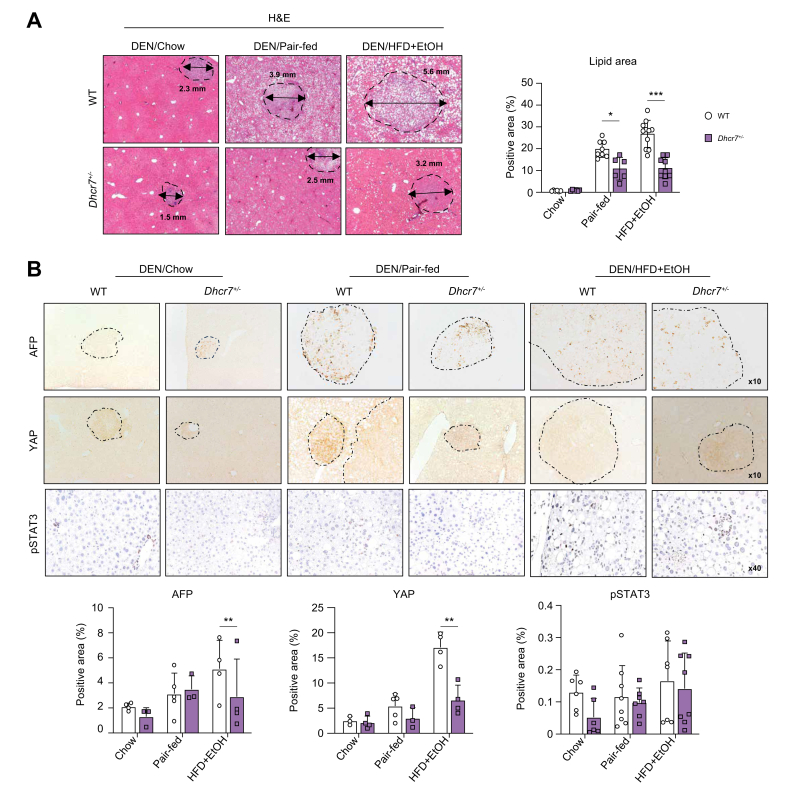
DEN/HFD + EtOH-fed *Dhcr7*^*+/–*^ mice are protected from HCC. (A) Livers were stained with H&E, and tumor diameters were measured. Representative images are show. Lipid area was calculated as a percentage. (B) Livers were stained for HCC markers AFP, YAP, and phospho-STAT3. Area of positive staining was calculated as a percentage. Data are mean ± SD; ∗*p* <0.05, ∗∗*p* <0.01, ∗∗∗*p* <0.001, two-tailed Student’s *t* test. DEN, diethylnitrosamine; DHCR7, 7-dehydrocholesterol reductase; EtOH, ethanol; HCC, hepatocellular carcinoma; HFD, high-fat diet; WT, wild-type.

H&E analysis of liver tissues revealed that hepatic steatosis was strongly suppressed (↓2.5-fold) in HFD + EtOH-fed *Dhcr7*^*+/–*^ mice (Fig. 3A). The tumor diameters were smaller (↓2-fold) in these mice than in WT mice. Interestingly, tumor diameters were also reduced in DEN/pair-fed and DEN/chow-fed *Dhcr7*^*+/–*^ mice, demonstrating that suppression of *Dhcr7* decreases HCC in steatotic and non-steatotic livers. Our data suggest that partial ablation of the *Dhcr7* gene was sufficient to attenuate the development of MetALD-induced steatosis and tumorigenesis in *Dhcr7*^*+/–*^ mice.

To determine the malignancy of liver tumors (HCC or adenomas), livers of DEN/HFD + EtOH-fed *Dhcr7*^*+/–*^ and WT mice were stained for the HCC-specific markers AFP and YAP and phospho-STAT3 (Fig. 3B). The majority (>83 ± 5%) of tumors from WT mice expressed AFP and YAP, indicating that alcohol-induced injury facilitates the development of HCC *vs*. adenomas. When HCC from DEN/HFD + EtOH-fed *Dhcr7*^*+/–*^ and WT mice were compared, expression of YAP was suppressed in *Dhcr7*^*+/–*^ mice, as shown by the reduced area of positive staining for YAP (↓3.0-fold), whereas expression of AFP and pospho-STAT3 was only slightly reduced in HCCs of DEN/ HFD + EtOH-fed *Dhcr7*^*+/–*^ mice. Unlike alcohol-injured mice, no significant difference in AFP, YAP, and pospho-STAT3 expression was observed in DEN/chow-fed *Dhcr7*^*+/–*^ and WT mice (Fig. 3B). Our data indicate that DHCR7 regulates malignancy and growth of metabolically injured hepatocytes in mice.

### Development of liver fibrosis was suppressed in DEN/HFD + EtOH-fed *Dhcr7*^*+/–*^ mice

**Fig. 4:**
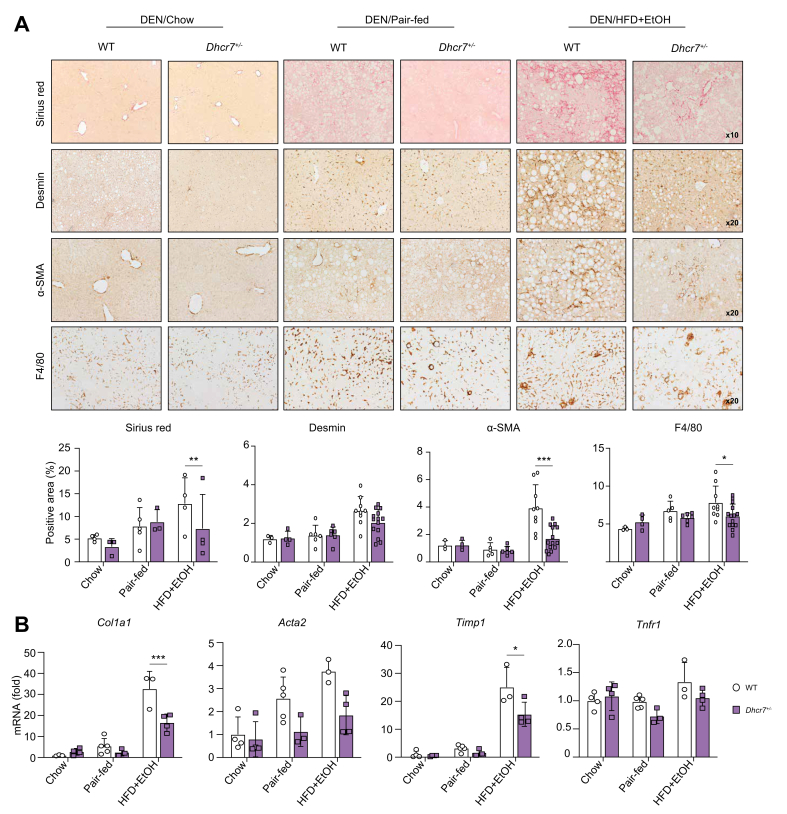
DEN/HFD + EtOH-fed *Dhcr7*^*+/–*^ mice develop less fibrosis and inflammation compared with WT littermates. (A) Livers were stained with Sirius red or immunostained for Desmin, αSMA, and F4/80. Area of positive staining was calculated as percent. Representative micrograph images were taken using 20× objectives. (B) Expression of fibrogenic and inflammatory genes was measured using qRT-PCR. Data are mean ± SD; ∗*p* <0.05, ∗∗*p* <0.01, ∗∗∗*p* <0.001, two-tailed Student’s *t* test. DEN, diethylnitrosamine; DHCR7, 7-dehydrocholesterol reductase; EtOH, ethanol; HFD, high-fat diet; qRT-PCR, quantitative real-time PCR; WT, wild-type.

Histological examination of alcohol-injured livers revealed that DEN/HFD + EtOH-fed WT mice developed marked fibrosis (↑3-fold) compared with DEN/pair-fed WT mice (which developed mild fibrosis) or DEN/chow-fed WT mice (which developed no fibrosis). In turn, DEN/HFD + EtOH-fed *Dhcr7*^*+/–*^ mice developed less fibrosis than WT mice, as shown by reduced positive area of Sirius red (↓2.5-fold), and αSMA staining (↓2.0-fold; Fig. 4A). This effect was associated with downregulation of fibrogenic genes *Col1a1, Acta2*, and *Timp1* (>↓1.5-fold; Fig. 4B), suggesting that DHCR7 regulates activation of myofibroblasts. Hepatic inflammation was slightly reduced in DEN/HFD + EtOH-fed *Dhcr7*^*+/–*^ mice based on downregulation of F4/80 expression in the livers of these mice, whereas expression of *Tnfr1* was not significantly different between DEN/HFD + EtOH-fed *Dhcr7*^*+/–*^ and WT mice (Fig. 4B). Meanwhile, partial DHCR7 deletion did not significantly change the expression of *Col1a1*, *Acta2*, and *Timp1* in DEN/pair-fed mice (Fig. 4B), mainly because of limited feeding time (18 weeks) to develop MASH. DEN/chow-fed *Dhcr7*^*+/–*^ and WT mice did not develop liver fibrosis.

### Pharmacological inhibition of DHCR7 activity lowered cholesterol levels in DEN/HFD + EtOH-fed WT mice

**Fig. 5:**
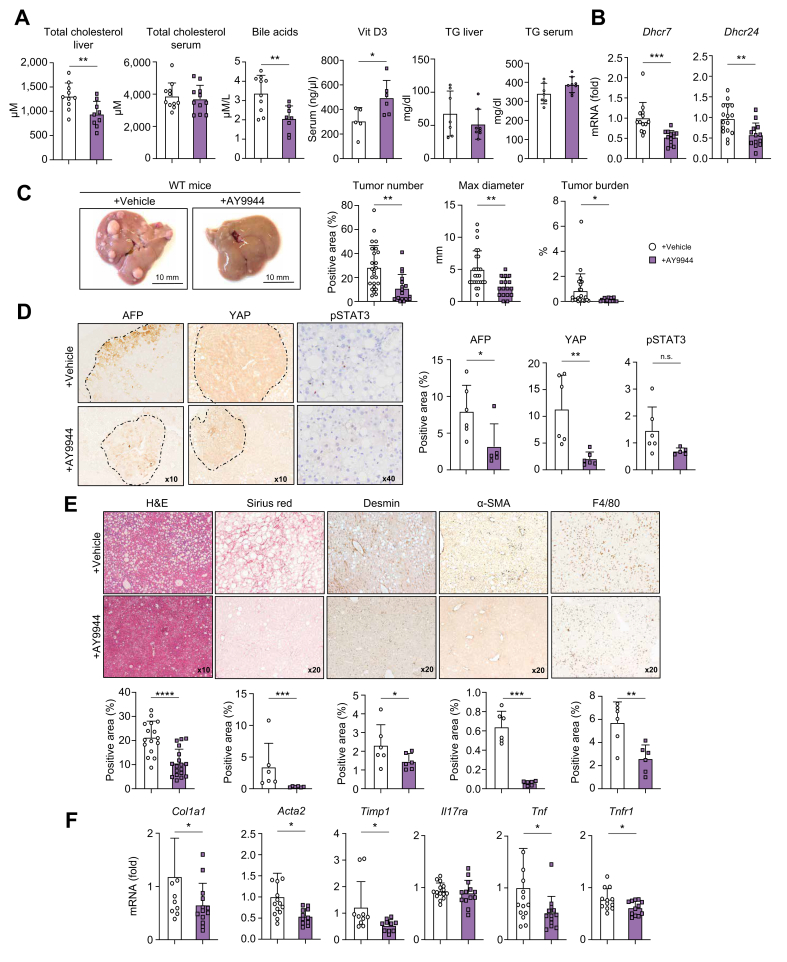
Pharmacological inhibition of DHCR7 inhibits MetALD and HCC in DEN/HFD + EtOH-fed WT mice. DEN/HFD + EtOH-fed WT mice (n ≥10 per group) were therapeutically administered ±AY9944 (10 mg/kg, i.p., three times a week) for the last 9 weeks of feeding. (A) Levels of total serum, hepatic cholesterol, serum bile acids, and vitamin D_3_ were measured. (B) Expression of *Dhcr7* and *Dhcr2*4 mRNA was measured using qRT-PCR. (C) Gross liver images of WT and *Dhcr7*^*+/–*^ mice. Tumor number, diameter, and burden were calculated. (D) Livers were stained for HCC markers AFP, YAP, and phospho-STAT3. (E) Livers were stained with H&E, Sirius red or immunostained for Desmin, αSMA, and F4/80. Area of positive staining was calculated as a percentage. (F) Expression of fibrogenic and inflammatory genes was measured using qRT-PCR. Data are mean ± SD; ∗*p* <0.05, ∗∗*p* <0.01, ∗∗∗*p* <0.001, two-tailed Student’s *t* test. DEN, diethylnitrosamine; DHCR7, 7-dehydrocholesterol reductase; EtOH, ethanol; HCC, hepatocellular carcinoma**;** HFD, high-fat diet; MetALD, metabolic dysfunction and alcohol-associated liver disease; qRT-PCR, quantitative real-time PCR; WT, wild-type.

We tested whether therapeutic inhibition of DHCR7 activity can attenuate the development of MetALD and HCC in mice. The DHCR7 inhibitor AY9944 was used to treat DEN/HFD + EtOH-fed WT mice, which have already developed significant liver injury (Fig. 5). Specifically, DEN/HFD + EtOH-fed WT mice were fed alcohol for 9 weeks and then administered AY9944 (10 mg/kg, i.p., three times a week) for the remaining 9 weeks of feeding, whereas control mice were injected with the vehicle. AY9944 inhibits the enzymatic activity of DHCR7, causing hypocholesterolemia. We observed a decrease (↓1.5-fold) in hepatic cholesterol and bile acid production in AY9944-treated DEN/ HFD + EtOH-fed WT mice (*vs*. control mice) and an increase (↑1.5-fold) in vitamin D_3_ levels (resulting from preferential conversion of 7-DHC into Vitamin D_3_ in the absence of DHCR7; Fig. 5A). Of note, the levels of triglycerides (not shown) and ferroptosis in HCC (Fig. S1) were not significantly changed in these mice. Because DHCR7 regulates DHCR24, expression of DHCR24 was also suppressed. Unexpectedly, expression of DHCR7 was also reduced (possibly via a feedback mechanism; Fig. 5B).

### AY9944-treated DEN/HFD + EtOH-fed WT mice were protected from HCC

Hepatic tumorigenesis was strongly suppressed in AY9944-treated DEN/HFD + EtOH-fed WT mice. The tumor size and tumor numbers were reduced (↓3.0 fold) compared with those in control mice (Fig. 5C). Tumor burden was reduced by ↓3-fold. Histopathological analysis revealed that expression of AFP, YAP, and phospho-STAT3 was downregulated in the livers of AY9944-treated DEN/HFD + EtOH-fed WT mice (Fig. 5D), indicating that blocking DHCR7 activity attenuates malignant transformation of metabolically injured hepatocytes.

### Development of MetALD was suppressed in AY9944-treated DEN/HFD + EtOH-fed WT mice

Lipid accumulation, liver fibrosis, and inflammation were markedly improved in AY9944-treated DEN/HFD + EtOH-fed WT mice (Fig. 5E). Specifically, hepatic steatosis was reduced by ↓2.0-fold in the livers of these mice. Hepatic fibrosis was improved, as shown by a reduced (↓4-fold) area of Sirius red positive staining. Activation of Desmin^+^ (↓1.8-fold) and αSMA^+^ (↓6-fold) myofibroblasts was suppressed (Fig. 5E), indicating that blocking DHCR7 activity prevents HSC activation and proliferation. Consistently, expression of fibrogenic markers *Col1a1*, *Acta2*, and *Timp1* was downregulated in AY9944-treated DEN/HFD + EtOH-fed WT mice (Fig. 5F). Hepatic inflammation was suppressed in AY9944-treated DEN/HFD + EtOH-fed WT mice and was associated with a reduced number (↓2-fold) of F4/80^+^ myeloid cells and downregulation of *Tnf* and *Tnfr1* in the livers of AY9944-treated DEN/HFD + EtOH-fed WT mice (Fig. 5F). Overall, our data demonstrate that therapeutic blocking of DHCR7 improves MetALD fibrosis and HCC.

### Therapeutic administration of AY9944 inhibitor attenuated growth of Hepa 1-6 HCC in the livers of HFD + EtOH-fed WT mice

**Fig. 6:**
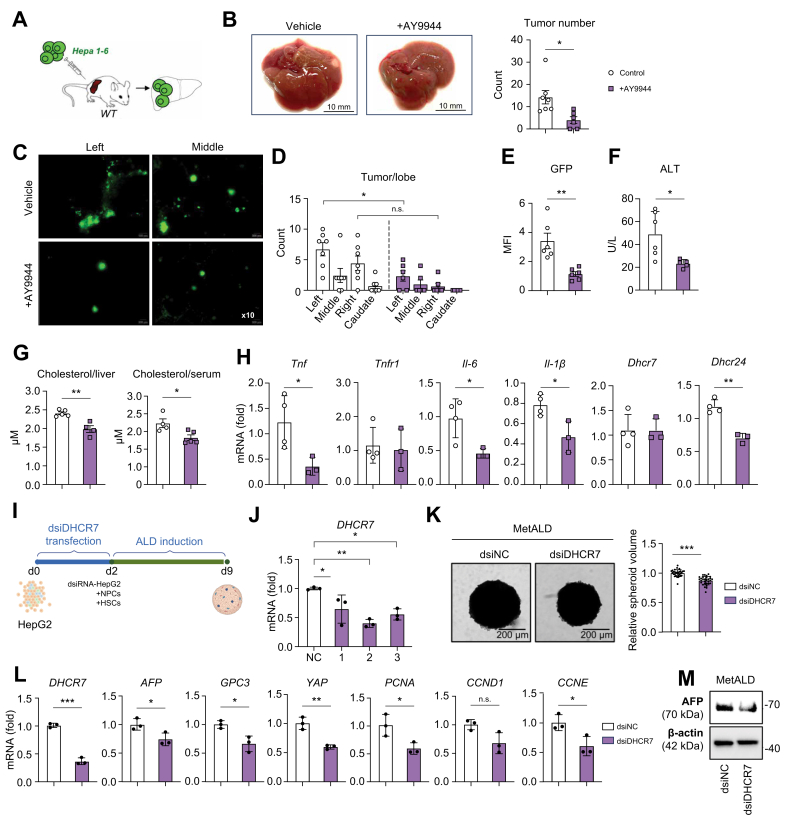
Administration of AY9944 inhibitor suppresses tumor growth in MetALD WT mice and MetALD-HCC human liver spheroids. (A) WT mice were fed HFD + EtOH for 2 weeks, adoptively transplanted with GFP^+^ Hepa1-6 cells (1 × 10^6^) via intrasplenic injection, followed by immediate splenectomy, and fed with HFD + EtOH for an additional 2 weeks. The AY9944 inhibitor was therapeutically administered (10 mg/kg, i.p., three times) during the last week of feeding. (B) Gross liver images of chimeric WT mice ± AY9944. Tumor number, diameter, and burden were calculated. (C) Representative liver images are shown, and GFP^+^ tumors were visualized using fluorescent microscopy. (D) The number of tumors per lobe was calculated. (E) GFP signal was calculated in the left lobe as mean fluorescent intensity. (F) Liver function was assessed by measuring serum levels of ALT. (G) Levels of total serum and hepatic cholesterol were measured. (H) Expression of inflammatory genes was measured by qRT-PCR. (I) MetALD-HCC human liver spheroids were generated using HepG2 cells (3 × 10^5^ cells transfected ± *DHCR7*-targeting dsiRNA), NPCs, and HSCs and cultured in a MetALD cocktail for 7 days. (J) dsiRNAs with three different hairpins were tested for *DHCR7* knockdown. (K) MetALD-HCC human liver spheroids (bright field images; scale bar = 200 μm). Relative volume of the HCC human liver spheroids was calculated as fold change compared with MetALD-HCC human liver spheroids containing HepG2 cells transfected with dsiRNA negative control. (L) Expression of HCC and proliferation markers was measured by qRT-PCR. (M) Expression of AFP was evaluated in MetALD-HCC human liver spheroids by Western blotting. Data are mean ± SD, ∗*p* <0.05, ∗∗*p* <0.01, ∗∗∗*p* <0.001, two-tailed Student’s *t* test. ALT, alanine aminotransferase; DEN, diethylnitrosamine; DHCR7, 7-dehydrocholesterol reductase; EtOH, ethanol; HCC, hepatocellular carcinoma; HFD, high-fat diet; HSC, hepatic stellate cell; MetALD, metabolic dysfunction and alcohol-associated liver disease; NPC, non-parenchymal cell; qRT-PCR, quantitative real-time PCR; WT, wild-type.

To further delineate the role of a cholesterol-rich environment on tumor growth, we examined whether AY9944 can inhibit hepatic growth of the mouse Hepa1-6 HCC cell line, adoptively transplanted into the livers of WT mice. WT mice were fed HFD + EtOH for 2 weeks and then injected with GFP-labeled Hepa 1-6 cells (1 × 10^6^) via intrasplenic injection (Fig. 6A), followed by immediate splenectomy.^20^ Mice continued to be fed HFD + EtOH for an additional 2 weeks. The AY9944 inhibitor was therapeutically administered (10 mg/kg, i.p., three times) into these mice during the last week of feeding. Gross liver images were evaluated using bright and fluorescent microscopy (Fig. 6B) to visualize GFP^+^ tumors, followed by histological analysis (Fig. 6C–E). The number of GFP^+^ tumors was calculated in the left, middle, and right caudate lobes (Fig. 6D). All mice engrafted Hepa 1-6 cells (Fig. 6C). The number of tumors per lobe was equally decreased in all liver lobes of AY9944-treated HFD + EtOH WT mice (*vs*. control mice; Fig. 6D). Consistently, tumor-derived fluorescent GFP signal was reduced in AY9944-treated HFD + EtOH chimeric WT mice (Fig. 6E), whereas liver function improved, as shown by downregulation of ALT levels (↓2.5-fold) in AY9944-treated HFD + EtOH WT mice (Fig. 6F). Hepatic and serum levels of cholesterol were slightly reduced in AY9944-treated HFD + EtOH chimeric WT mice (Fig. 6G). Expression of *Dhcr7* remained unchanged, whereas expression of *Dhcr24* was reduced. Moreover, the level of inflammatory cytokines (*Il-1β*, *Il-6*, and *Tnf*) was also reduced in AY9944-treated HFD + EtOH chimeric WT mice (Fig. 6H). Our data suggest that suppression of cholesterol activity in the tumor microenvironment inhibits HCC growth.

### Knockdown of *DHCR7* suppresses HCC progression in HCC human liver spheroids with MetALD

The role of DHCR7 in tumorigenesis was tested using MetALD-HCC human liver spheroids (Fig. 6I). For this purpose, human HCC cell line HepG2 cells were transfected with *DHCR7*-targeting or scrambled (negative control) dsiRNAs. Three different hairpins were tested. The dsiRNA with the highest knockdown efficiency (>60%, dsiDHCR7-2) was selected (Fig. 6J) before spheroid formation. Spheroids containing DHCR7-knocked down or control HepG2 (3 × 10^5^), human NPCs (1.5 × 10^5^), and HSCs (0.8 × 10^5^) were cultured in a MetALD cocktail for 7 days. Proliferation of MetALD-HCC spheroids containing *DHCR7*-knocked down HepG2 cells was significantly suppressed, as shown by reduced spheroid volume (Fig. 6K) and downregulation of cell proliferation markers, *PCNA, CCND1*, and *CCNE1* (Fig. 6K and L). Moreover, expression of HCC markers *AFP*, *GPC3*, and *YAP1* was also suppressed in *DHCR7*-knocked down MetALD-HCC human liver spheroids (Fig. 6L and M). Overall, our data indicate that targeted downregulation of *DHCR7* expression in human HCC or inhibition of *DHCR7* activity in the tumor microenvironment can ameliorate the development of MetALD and HCC.

### Generation of 3D human liver spheroids with MetALD

**Fig. 7:**
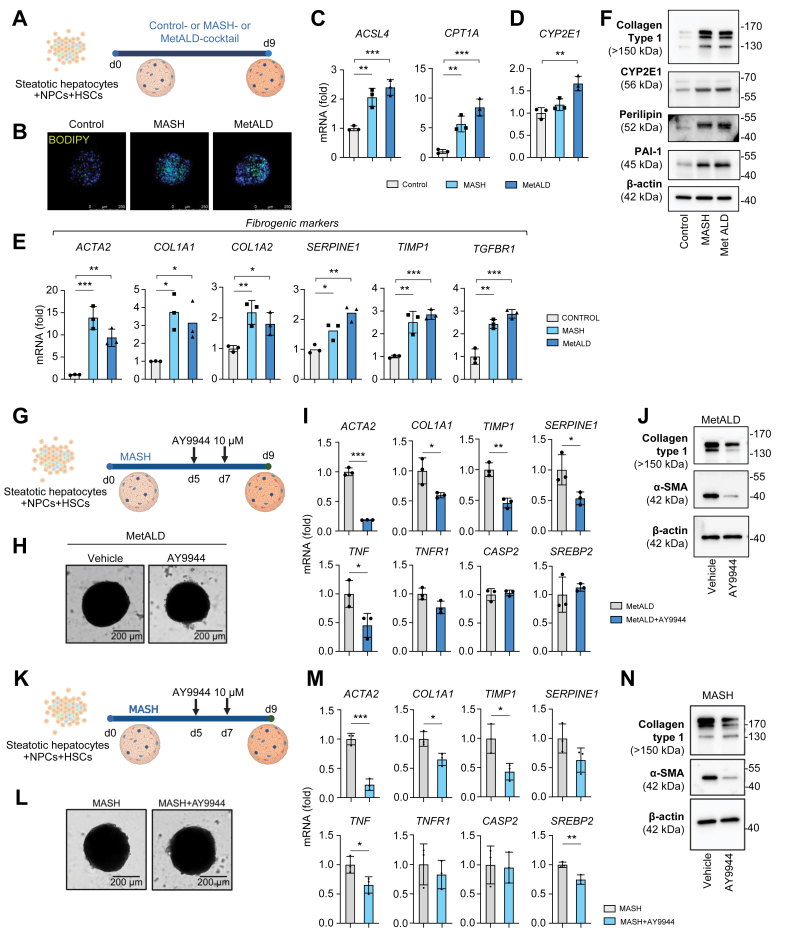
Inhibition of DHCR7 suppresses the development of MASH and MetALD in human liver spheroids. (A) MASH- or MetALD-induced human liver spheroids were generated using primary steatotic hepatocytes, NPCs, and HSCs, and cultured in MASH or MetALD cocktails for 9 days ± AY9944 (administered during the last 4 days of culture). (B) Micrographs represent human liver spheroids stained with BODIPY and DAPI (scale bar = 250 μm). mRNA expression of (C) fatty acid-modifying enzymes, (D) CYP2E1, or (E) fibrogenic markers in MASH or MetALD human liver spheroids (*vs*. control human liver spheroids). (F) Protein expression of fibrogenic markers in MASH or MetALD human liver spheroids detected by Western blotting. (G) Outline of AY9944 treatment of human liver spheroids with MetALD. (H) MetALD human liver spheroids (representative bright field images; scale bar = 200 μm). Expression of fibrogenic genes in AY9944-treated MetALD spheroids was measured by (I) qRT-PCR or (J) Western blotting. (K) Outline of AY9944 treatment of human liver spheroids with MASH. (L) MASH human liver spheroids (representative bright field images; scale bar = 200 μm). Expression of fibrogenic genes in AY9944-treated MASH spheroids was measured by (M) qRT-PCR or (N) Western blotting. DHCR7, 7-dehydrocholesterol reductase; HSC, hepatic stellate cell; MASH, metabolic dysfunction-associated steatohepatitis; MetALD, metabolic dysfunction and alcohol-associated liver disease; NPC, non-parenchymal cell; qRT-PCR, quantitative real-time PCR.

To translate our findings, the role of DHCR7 was assessed using human liver spheroids with MetALD. Human liver spheroids were generated by co-culturing all liver cell types, which included freshly isolated primary human steatotic hepatocytes (3 × 10^5^) + NPCs (1.5 × 10^5^) + HSCs (0.8 × 10^5^), at physiological ratios in the presence of MASH cocktail (160 μM palmitate, 160 μM oleate, 10 mM fructose, 5.5 mM glucose, 10 μg/ml LPS, and 1 ng/ml TGFβ1) or MetALD cocktail (MASH cocktail + 100 mM of ethanol) for 9 days (Fig. 7A).^23^^,^^24^ The effect of alcohol on the development of steatosis, inflammation, and fibrosis was assessed. Exposure to alcohol caused significant upregulation of lipid droplets (BODIPY), lipid-metabolizing enzyme (*ACSL4*) and rate-limiting step of fatty acid oxidation (*CPT1A*), detoxifying enzyme (*CYP2E1*), and markers of HSC activation (*SERPINE1*, *TIMP1*, and *TGFBR1*) in MetALD human liver spheroids to levels higher than those in MASH spheroids. Expression of fibrogenic genes (*ACTA2*, *COL1A1*, and *COL1A2*) was slightly lower in MetALD spheroids than in MASH spheroids (Fig. 7B–F). We conclude that human liver spheroids with MetALD recapitulate alcohol-induced injury of human livers.

### Inhibition of DHCR7 activity attenuates the development of MetALD and MASH in 3D human liver spheroids

The role of cholesterol in the development of liver fibrosis and tumorigenesis was studied using MetALD and MetALD-HCC spheroids, respectively.

First, we tested whether therapeutic inhibition of DHCR7 activity can attenuate the development of MetALD in the human liver spheroids. At Days 5 and 7, human liver spheroids were treated with ±AY9944 (10 μM; Fig. 7G and H). Fibrogenic genes (*ACTA2*, *COL1A1*, *TIMP1*, and *SERPINE1*) and inflammatory genes (*TNF*) were significantly reduced in AY9944-treated MetALD spheroids (Fig. 7I). Expression of *DHCR7* regulatory molecules, *SREBP2* and *Caspase2*, was not changed, indicating inhibition of DHCR7 activity did not activate compensatory upregulation of lipogenic enzymes (Fig. 7I). Remarkably, expression of collagen type I and αSMA mRNA and proteins was strongly reduced in AY9944-treated MetALD spheroids (*vs*. MetALD spheroids; Fig. 7J).

Similarly, we assessed whether therapeutic inhibition of DHCR7 activity can attenuate the development of MASH in the human liver spheroids. MASH spheroids were treated with ±AY9944 (10 μM; Fig. 7K and L). Fibrogenic genes (*ACTA2*, *COL1A1*, and *TIMP1*) and inflammatory genes (*TNF*) were significantly reduced in AY9944-treated MASH spheroids (Fig. 7M). Expression of collagen type I and αSMA proteins was strongly reduced in AY9944-treated MASH spheroids (*vs*. MASH spheroids; Fig. 7N), indicating that suppression of cholesterol synthesis ameliorates MetALD and MASH.

## Discussion

Cholesterol homeostasis is essential for health. However, excessive cholesterol synthesis promotes metabolic liver injury and HCC.^25^ The role of the cholesterol biosynthetic enzyme DHCR7 in the pathogenesis of MetALD is not well understood. Our study investigates the effect of DHCR7-dependent *de novo* lipogenesis in preclinical models of MetALD and HCC. We demonstrated that genetic and pharmacological inhibition of DHCR7 suppresses steatosis, inflammation, fibrosis, and HCC in DEN-challenged HFD + EtOH-fed mice or 3D human liver spheroids, which mimic “MetALD in a dish.” Reducing DHCR7 expression and activity in tumors or the tumor microenvironment ameliorated MetALD, suggesting that targeting *cholesterol synthesis* can become a novel strategy for the treatment of MetALD and HCC.

We used *Dhcr7*^*+/–*^ mice, which carry one copy of the *Dhcr7* gene, to study the effect of DHCR7-dependent *de novo* lipogenesis in MetALD. Chronic exposure to alcohol strongly accelerated liver injury and HCC in DEN/HFD + EtOH-fed WT (*vs*. pair-fed mice). Remarkably, *Dhcr7*^*+/–*^ mice were protected from alcohol-induced hepatic steatosis, inflammation, fibrosis, and HCC (*vs*. WT mice). Partial *Dhcr7* deletion produced the most profound effect on tumorigenesis in DEN/HFD + EtOH-fed *Dhcr7*^*+/–*^ mice, as shown by reduced tumor number, diameter, and expression of AFP and YAP, and phospho-STAT3. Partial *Dhcr7* deletion also ameliorated alcohol-induced liver injury. In addition, partial ablation of *Dhcr7* was shown to attenuate acetaminophen-induced liver injury in mice.^10^ These findings suggest that DHCR7 can serve as a potential target for the treatment of metabolic liver diseases caused by dysregulation of cholesterol synthesis.

Maintaining cholesterol homeostasis is achieved by balancing cholesterol synthesis, dietary absorption, and excretion. *De novo* synthesis (∼70%) is the primary source of cholesterol in humans, with dietary intake contributing to ∼30%.^26^ The liver produces the majority of the *de novo* synthesized cholesterol. Excessive accumulation of cholesterol exerts a strong hepatotoxic effect and promotes HCC.^22^ Several classes of drugs were generated to target *de novo* lipogenesis and cholesterol synthesis, such as HMGCR inhibitors,^27^^,^^28^ inhibitors of S1P (an enzyme that critically regulates SREBP1/2 activity and transcription of target genes, including DHCR7), and inhibitors of DHCR7. Statins produce strong cholesterol-lowering effects and mediate anti-inflammatory, antifibrotic, and antiproliferative effects in the liver.^25^ The S1P inhibitor PF-429242 can lower cholesterol synthesis by preventing SREBP1/2 activation.^29^ The DHCR24 inhibitor genkwadaphnin (GD) can effectively suppress DHCR24-mediated cholesterol metabolism and prevent HCC growth in xenograft models.^30^ The DHCR7 inhibitor AY9944 [trans-1,4-bis(2-chlorobenzylaminomethyl) cyclohexane dihydrochloride] suppresses ferroptosis and ischemia–reperfusion injury^10^ but has not been evaluated for the treatment of MetALD.

We tested the effect of the DHCR7 inhibitor using novel models of 3D MetALD human liver spheroids. Our data demonstrate that treatment of human MetALD liver spheroids significantly reduces steatosis, inflammatory responses, and fibrogenic activation of myofibroblasts, suggesting that AY9944 is suitable for MetALD therapy. However, the long-term use of the AY9944 inhibitor can elicit side effects, such as depletion of cholesterol.^10^ Generation of new classes of DHCR7 inhibitors is needed. Alternatively, DHCR7 expression can be decreased using siRNA-based approaches. Our study demonstrated that blocking DHCR7 in the human HCC cell line HepG2 attenuated tumor growth and malignization, indicating that inhibition of DHCR7 in the tumor and/or the tumor microenvironment can produce a strong therapeutic effect on alcohol-induced liver injury and HCC.

Overall, our study underlines the critical role of DHCR7 in the pathogenesis of MetALD and HCC. Future studies will elucidate the contribution of DHCR7-cholesterol synthesis across different types of liver cancer and investigate the therapeutic efficacy of DHCR7 inhibitors administered in combination with other treatments.

## Abbreviations

ALT, alanine aminotransferase; DEN, diethylnitrosamine; DHCR7, 7-dehydrocholesterol reductase; EtOH, ethanol; GFP, green fluorescent protein; HCC, hepatocellular carcinoma; HFD, high-fat diet; HSC, hepatic stellate cell; MASH, metabolic dysfunction-associated steatohepatitis; MetALD, metabolic dysfunction and alcohol-associated liver disease; MFI, mean fluorescence intensity; NPC, non-parenchymal cell; OS, overall survival; qRT-PCR, quantitative real-time PCR; RNA-seq, RNA sequencing; WT, wild-type.

## Financial support

This research was supported by the 10.13039/100000002National Institutes of Health R01DK111866, R56DK088837, DK099205, AA028550, DK101737, AA011999, DK120515, AA029019, DK091183, P42ES010337, R44DK115242 (TK and DAB), R01CA285997 (DAB), and by Sanford Stem Cell Fitness and Space Medicine Center at Sanford Stem Cell Institute (UCSD, TK), Shanghai Pujiang Program (21PJD027) (NL).

## Authors’ contributions

Performed experiments: GY, RCGW, VZ, HJ, SD, XL, HYK. Analyzed the data: GY, RCGW. Designed the study, provided support, and wrote the manuscript: DAB, NL, TK.

## Data availability statement

The data supporting the findings of this study are available within the article and its Supplemental materials.

## Conflicts of interest

The authors have nothing to declare.

Please refer to the accompanying ICMJE disclosure forms for further details.
