# Supplementary material for: DHCR7 inhibition ameliorates MetALD and HCC in mice and human 3D liver spheroids
Source: JHEP Rep. 2025 Apr 5;7(7):101415. doi: 10.1016/j.jhepr.2025.101415 (PMC12173064; doi:10.1016/j.jhepr.2025.101415)
Supplement: Multimedia component 1 [file mmc1.pdf]

DHCR7 inhibition ameliorates MetALD and HCC in mice and human 3D liver spheroids

Gen Yamamoto, Raquel Carvalho-Gontijo Weber, Wonseok Lee, Vivian Zhang, Haeum Jang, Sadatsugu Sakane, Xiao Liu, Hyun Young Kim, David A. Brenner, Na Li, Tatiana Kisseleva

Table of contents

Supplementary methods…………………………………………………………….……….2

Supplementary text…………………………………………………………………….. 2

Fig. S1……………………………………………………..…………………………..3

Table S1……………………………………………………………………………………4

Supplementary references…………………………………………..…………………4

Supplementary methods

Cell viability assay: Cell viability was measured using the WST-1 assay reagent (Abcam). HepG2 cells were plated in 96-well plates at a density of 8,000 per well and treated with erastin (MedChem), ferrostatin-1 (MedChem), AY9944 (Cayman) or their combination for 24 h. Next, 10 uL of WST-1 assay reagent was added to each well and incubated for 1 h. Cell viability was assessed by measuring the absorbance at 450 nm using SpectraMax iD3 plate reader (Molecular Devices).

Western Blot Analysis: Mouse liver tumors were harvested and lysed in RIPA buffer containing protease and phosphatase inhibitor cocktail (Sigma). The proteins were separated by SDS-PAGE and transferred to polyvinylidene difluoride membranes. The membranes were blocked in 5% nonfat milk and incubated with the indicated primary antibodies. After incubation with secondary antibodies, proteins were detected using Supersignal West Pico Plus chemiluminescence substrate (Thermo Scientific). The primary antibodies and dilutions were as follows: β-actin (A5441, Sigma-Aldrich, 1:5,000), ACSL4 (PA5-27137, Invitrogen, 1:1,000), FTH1 (4393, Cell Signaling, 1:2,000) GPX4 (14432-1-AP, Proteintech, 1:2,000). Membranes were washed and exposed to HRP-conjugated secondary antibodies for 1 hour at room temperature. Protein was detected by the ECL Plus Western blotting detection system (BioRad) and was visualized by the BioRad Chemidoc Touch Imaging System.

Supplementary text

Ferroptosis is an iron-dependent form of regulated cell death that occurs as a consequence of lethal lipid peroxidation. *GPX4* converts potentially toxic lipid hydroperoxides (L-OOH) to non-toxic lipid alcohols (L-OH), and decrease of *GPX4* results in increased lipid peroxidation that causes ferroptosis[1]. Ferroptosis is implicated in the pathogenesis of various liver diseases. Inhibition of *DHCR7* was shown to suppress ferroptosis by 7-DHC functions as a radical trapping agent[2, 3].

To investigate whether the *DHCR7* inhibitor AY9944 suppresses HCC development through ferroptosis inhibition in DEN/HFD+EtOH-injured wild type mice, we tested the effect of well known ferroptosis inducer, erastin, and a ferroptosis inhibitor, ferrostatin-1 in cultured HepG2 cells. Erastin significantly induced ferroptosis-mediated cell death in HepG2 cells as shown by decreased cell viability. Ferroptosis-induced cell death was reduced, when erastin-treated HepG2 cells were pre-treated with ferrostatin-1 (Supplemental Figure 1A). Meanwhile, pre-treatment of with AY9944 did not reverse ferroptosis-induced apoptosis of HepG2 cells (Supplemental Figure 1B). Furthermore, HCC from DEN/HFD+EtOH-injured wild type mice ± AY9944 were analyzed for expression of ferroptosis markers. Ferroptosis is accompanied by decreased activity of glutathione peroxidase (GPX4) and ferritin heavy chain 1 (FTH1), and increased expression of acyl-CoA synthetase long-chain family 4 (ACSL4)[1]. Ferroptosis markers *ACSL4*, *FTH1*, and *GPX4* protein expressions were not significantly changed between AY9944- and vehicle-treated DEN/HFD+EtOH-injured wild type mice (Supplemental Figure 1C). These results demonstrate that ferroptosis inhibition is not the main mechanism contributing to the effect of AY9944 on HCC suppression.

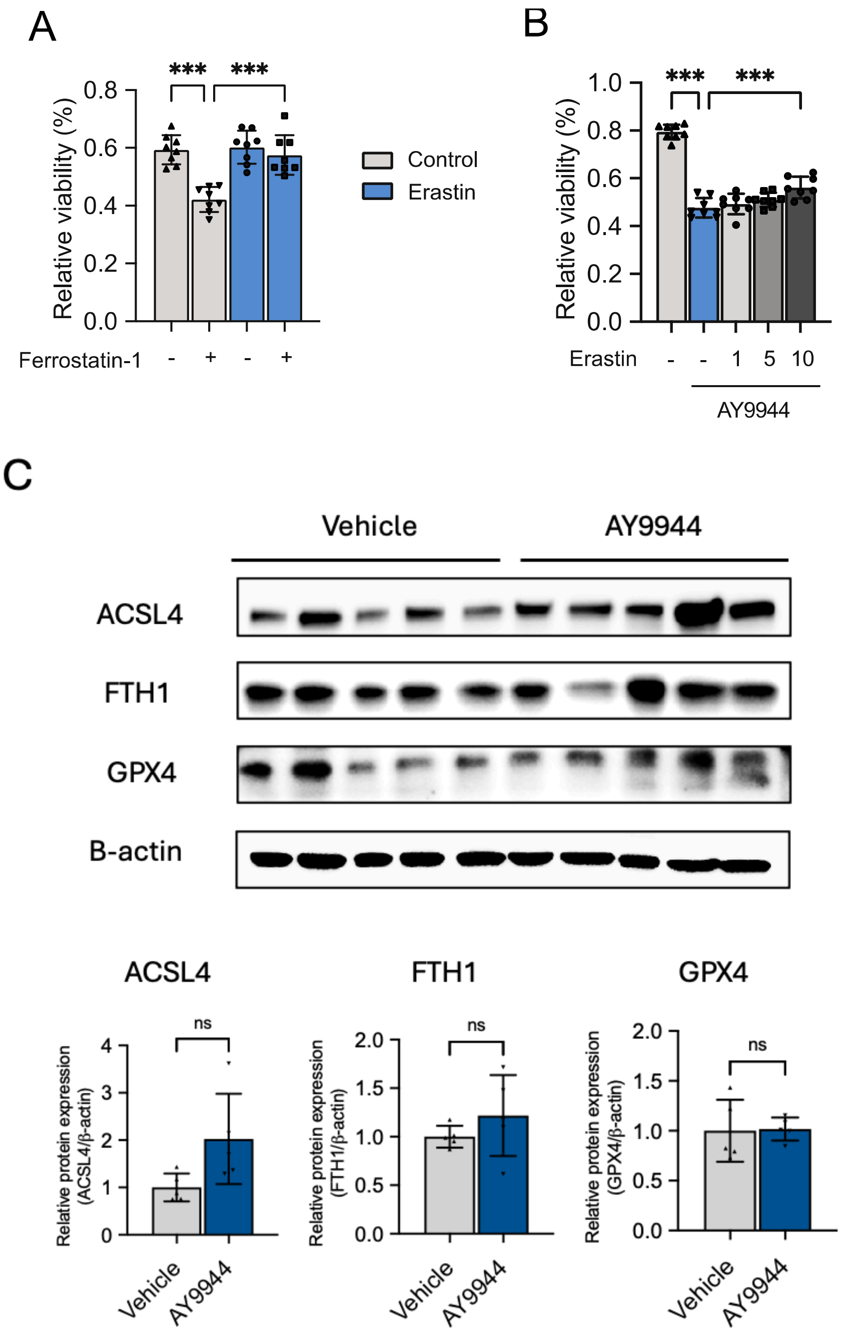

Fig. S1. Inhibition of *DHCR7* with AY9944 inhibitor minimally affects ferroptosis in DEN/HFD+EtOH-injured mice. (A) HepG2 cells were pretreated with ferrostatin-1 (5 μM) for 1 h and then treated with erastin (10 μM) for 24 h. Cell viability was assessed by the WST-1 assay. (B) HepG2 cells were pretreated with AY9944 (10 μM) for 1 h and then treated with erastin (10 μM) for 24 h. Cell viability was assessed by the WST-1 assay. Data are mean ± SD (n = 8); ****p* <0.001, one-way ANOVA followed by Tukey's test. (C) Protein expression of *ASCL4, FTH1*, and *GPX4* was assessed by western blotting of tumors from WT mice treated with AY9944 or vehicle control (n=5 per group). Densitometric quantification was performed with Image J. Data are

presented as mean ± SD. **p <0.01 and ns, not significant, 2-tailed Student's t-test. (Supporting Figure 5)

Table S1. OS analysis of clinicopathological characteristics and DHCR7 expression in HCC patients using Kaplan-Meier Plotter database.

Features	Variables	Number of Patients	Number of patient at risk	Median Survival (months)	Logrank P	HR
	Stage 1	170	Low117	84.4		1.23(0.65-2.34)
			high 53	71	0.53	
Pathology	Stage 2	83	low 60	108.6		3.13(1.38-7.12)
			high 23	20	0.0042	
	Stage 3	83	low 29	18.5		0.74(0.39-1.41)
			high 54	27.6	0.36	
	Hepatitis virus: Yes	150	low 52	23.1		0.6 (0.32-1.15)
Risk factor			high 98	54.1	0.12	
	Hepatitis virus: No	167	low 87	47.4		1.59(1.01-2.52)
			high 80	27.9	0.045	

Legend: The table describes the relationship between pathological features, DHCR7 expression levels, and survival probability.

Supplementary references

[1] Stockwell BR, Friedmann Angeli JP, Bayir H, Bush AI, Conrad M, Dixon SJ, et al. Ferroptosis: A Regulated Cell Death Nexus Linking Metabolism, Redox Biology, and Disease. Cell 2017;171:273-285.

[2] Freitas FP, Alborzinia H, dos Santos AF, Nepachalovich P, Pedrera L, Zilka O, et al. 7-Dehydrocholesterol is an endogenous suppressor of ferroptosis. Nature 2024;626:401-410.

[3] Yamada N, Karasawa T, Ito J, Yamamuro D, Morimoto K, Nakamura T, et al. Inhibition of 7-dehydrocholesterol reductase prevents hepatic ferroptosis under an active state of sterol synthesis. Nature Communications 2024;15:2195.
