# Supplementary material for: DHCR7 inhibition ameliorates MetALD and HCC in mice and human 3D liver spheroids
Source: JHEP Rep. 2025 Apr 5;7(7):101415. doi: 10.1016/j.jhepr.2025.101415 (PMC12173064; doi:10.1016/j.jhepr.2025.101415)
Supplement: Multimedia component 2 [file mmc2.docx]

**JHEP Reports**

**CTAT methods**

Tables for a “Complete, Transparent, Accurate and Timely account” (CTAT) are now mandatory for all revised submissions. The aim is to enhance the reproducibility of methods.

- Only include the parts relevant to your study
- Refer to the CTAT in the main text as ‘Supplementary CTAT Table’
- Do not add subheadings
- Add as many rows as needed to include all information
- Only include one item per row

**If the CTAT form is not relevant to your study, please outline the reasons why:**

|  |
| --- |

- 1. **Antibodies**

| **Name** | **Citation** | **Supplier** | **Cat no.** | **Clone no.** |
| --- | --- | --- | --- | --- |
| **anti-α-SMA** | **J Clin Invest. 2017;127(4):1254–1270** | **Abcam** | **ab5694** | **Polyclonal** |
| **anti-Desmin** | **Nat Commun. 2013;4:2823** | **Thermo Fisher Scientific** | **RB-9014-P0** | **Polyclonal** |
| **anti-F4/80** | **J Clin Invest. 2017;127(4):1254–1270** | **eBioscience** | **14-4801-88** | **BM8** |
| **anti-AFP** | **Anticancer Res. 2012;32(11):4987-4991.** | **Biocare Medical** | **CP028A** | **Polyclonal** |
| **Anti-AFP** | **Nat Commun**  **2024 29;15(1):1879.** | **Cell Signalling Technology** | **4448S** | **D12C1** |
| **Anti-YAP** | **Cell. 2012;150(4), 780-791.** | **Cell Signalling Technology** | **8418** | **D24E4** |
| **Anti-pSTAT3** | **Nat Commun. 2019;10(1):3601.** | **Cell Signalling Technology** | **9145** | **D3A7** |
| **Anti-β-actin** | ***Nat Commun 2019, 10(1), 3601.*** | **Sigma-Aldrich** | **A5441** | **AC-15** |
| **Anti-Collagen Type 1** | ***Nat Commun***  ***2024, 15(1):5449*** | **Cell Signalling Technology** | **72026S** | **E8F4L** |
| **Anti- CYP2E1** | **Molecular immunology,**  **2016, 75, 122.** | **Millipore** | **AB1252** | **Polyclonal** |
| **Anti- Perilipin 2** | **Hepatology Commun**  **2023, 7(11)** | **Novus Biologicals** | **NB110-40877** | **Polyclonal** |

- 1. **Cell lines**

| **Name** | **Citation** | **Supplier** | **Cat no.** | **Passage no.** | **Authentication test method** |
| --- | --- | --- | --- | --- | --- |
| **HEPA 1-6** | **Cell Reports Medicine 5**  **2024, 101718** | **ATCC, Manassas** | **CRL-1830** |  |  |
| **HepG2** | ***Cancer Reports,* 2024; 7:e2161** | **ATCC, Manassas** | **HB-8065** |  |  |

- 1. **Organisms**

| **Name** | **Citation** | **Supplier** | **Strain** | **Sex** | **Age** | **Overall n number** |
| --- | --- | --- | --- | --- | --- | --- |
| **Dhcr7^+/-^** | **Mol Genet Metab**  **2007, 91(1):7-14** | **Jackson Laboratories** | **C56BL/6** | **Male** | **14 days old; 12 weeks old** | **20** |

- 1. **Sequence based reagents**

| **Name** | **Sequence** | **Supplier** |
| --- | --- | --- |
| **Human** *HPRT* | Forward Primer (5ʹ to 3ʹ) CCTGGCGTCGTGATTAGTGAT  Reverse Primer (5ʹ to 3ʹ)  AGACGTTCAGTCCTGTCCATAA | **IDT** |
| **Human** *DHCR7* | Forward Primer (5ʹ to 3ʹ) GCTGCAAAATCGCAACCCAA  Reverse Primer (5ʹ to 3ʹ)  GCTCGCCAGTGAAAACCAGT | **IDT** |
| **Human** *CASPASE2* | Forward Primer (5ʹ to 3ʹ)  AGCTGTTGTTGAGCGAATTGT  Reverse Primer (5ʹ to 3ʹ)  AGCAAGTTGAGGAGTTCCACA | **IDT** |
| **Human** *ACSL4* | Forward Primer (5ʹ to 3ʹ)  TTTGCTGCCTGTCCACTTGTT  Reverse Primer (5ʹ to 3ʹ)  AGGTTTGTCTGAAGTGGGCT | **IDT** |
| **Human** *CPT1A* | Forward Primer (5ʹ to 3ʹ)  GCAGCGTTCTTTGTGACGTT  Reverse Primer (5ʹ to 3ʹ)  AGGAGTGTTCAGCGTTGAGG | **IDT** |
| **Human** *CYP2E1* | T Forward Primer (5ʹ to 3ʹ)  TGAAGCCTCTCGTTGACCC  Reverse Primer (5ʹ to 3ʹ)  CGTGGTGGGATACAGCAA | **IDT** |
| **Human** *ACTA2* | Forward Primer (5ʹ to 3ʹ)  CACCATCGGAAATGAACGTTT  Reverse Primer (5ʹ to 3ʹ)  GACTCCATCCCGATGAAGGA | **IDT** |
| **Human** *COL1A1* | Forward Primer (5ʹ to 3ʹ)  AAGAGGAAGGCCAAGTCGAG  Reverse Primer (5ʹ to 3ʹ)  CACACGTCTCGGTCATGGTA | **IDT** |
| **Human** *COL1A2* | Forward Primer (5ʹ to 3ʹ)  CCGTGCTTCTCAGAACATCA  Reverse Primer (5ʹ to 3ʹ)  CTTGCCCCATTCATTTGTCT | **IDT** |
| **Human** *SERPINE1* | Forward Primer (5ʹ to 3ʹ)  AGTGGACTTTTCAGAGGTGGA  Reverse Primer (5ʹ to 3ʹ)  GCCGTTGAAGTAGAGGGCATT | **IDT** |
| **Human** *SREBP2* | Forward Primer (5ʹ to 3ʹ)  AACGGTCATTCACCCAGGTC  Reverse Primer (5ʹ to 3ʹ)  GGCTGAAGAATAGGAGTTGCC | **IDT** |
| **Human** *TIMP1* | Forward Primer (5ʹ to 3ʹ)  CTTCTGCAATTCCGACCTCGT  Reverse Primer (5ʹ to 3ʹ)  ACGCTGGTATAAGGTGGTCTG | **IDT** |
| **Human** *TGFBR1* | Forward Primer (5ʹ to 3ʹ)  TCCAACTACTGGTTTACCATTGC  Reverse Primer (5ʹ to 3ʹ)  CCGCCACTTTCCTCTCCAAA | **IDT** |
| **Human** *TNFR1* | Forward Primer (5ʹ to 3ʹ)  TCACCGCTTCAGAAAACCACC  Reverse Primer (5ʹ to 3ʹ)  GGTCCACTGTGCAAGAAGAGA | **IDT** |
| **Human** *TNF* | Forward Primer (5ʹ to 3ʹ)  TGCACTTTGGAGTGATCGGC  Reverse Primer (5ʹ to 3ʹ)  CTCAGCTTGAGGGTTTGCTAC | **IDT** |
| **Human** *AFP* | Forward Primer (5ʹ to 3ʹ)  GCAGAGGAGATGTGCTGGATTG  Reverse Primer (5ʹ to 3ʹ)  CGTGGTCAGTTTGCAGCATTCTG | **IDT** |
| **Human** *GPC3* | Forward Primer (5ʹ to 3ʹ)  CATTGGAGGCTCTGGTGATGGA  Reverse Primer (5ʹ to 3ʹ)  TTGTCCTTCGGAGTTGCCTGCT | **IDT** |
| **Human** *YAP1* | Forward Primer (5ʹ to 3ʹ)  AGCAGGATGGTGGGACTCAAAAT  Reverse Primer (5ʹ to 3ʹ)  AGGTGCCACTGTTAAGGAAAGGAT | **IDT** |
| **Human** *PCNA* | Forward Primer (5ʹ to 3ʹ)  CAAGTAATGTCGATAAAGAGGAGG  Reverse Primer (5ʹ to 3ʹ)  GTGTCACCGTTGAAGAGAGTGG | **IDT** |
| **Human** *CCND1* | Forward Primer (5ʹ to 3ʹ)  TCTACACCGACAACTCCATCCG  Reverse Primer (5ʹ to 3ʹ)  TCTGGCATTTTGGAGAGGAAGTG | **IDT** |
| **Human** *CCNE1* | Forward Primer (5ʹ to 3ʹ)  TGTGTCCTGGATGTTGACTGCC  Reverse Primer (5ʹ to 3ʹ)  CTCTATGTCGCACCACTGATACC | **IDT** |
| **Mouse** *Hprt* | Forward Primer (5ʹ to 3ʹ)  GTTAAGCAGTACAGCCCCAAA  Reverse Primer (5ʹ to 3ʹ)  AGGGCATATCCAACAACAAACTT | **IDT** |
| **Mouse** *Dhcr7* | Forward Primer (5ʹ to 3ʹ)  GCTCTTCGCTCCATTCATTGT  Reverse Primer (5ʹ to 3ʹ)  AAGCCAGGAATAAAGCAGCAC | **IDT** |
| **Mouse** *Srebp1* | Forward Primer (5ʹ to 3ʹ)  GCAGCCACCATCTAGCCTG  Reverse Primer (5ʹ to 3ʹ)  CAGCAGTGAGTCTGCCTTGAT | **IDT** |
| **Mouse** *Srebp2* | Forward Primer (5ʹ to 3ʹ)  GCAGCAACGGGACCATTCT  Reverse Primer (5ʹ to 3ʹ)  CCCCATGACTAAGTCCTTCAACT | **IDT** |
| **Mouse** *Tnfr1* | Forward Primer (5ʹ to 3ʹ)  CCGGGAGAAGAGGGATAGCTT  Reverse Primer (5ʹ to 3ʹ)  TCGGACAGTCACTCACCAAGT | **IDT** |
| **Mouse** *Il-17ra* | Forward Primer (5ʹ to 3ʹ)  CTTGACTCTGCAGCTCAGCC  Reverse Primer (5ʹ to 3ʹ)  ATGGCTGCTTCTGCTGCT | **IDT** |
| **Mouse** *Il-6* | Forward Primer (5ʹ to 3ʹ)  ACCAGAGGAAATTTTCAATAGGC  Reverse Primer (5ʹ to 3ʹ)  TGATGCACTTGCAGAAAACA | **IDT** |
| **Mouse** *Col1a1* | Forward Primer (5ʹ to 3ʹ)  TAGGCCATTGTGTATGCAGC  Reverse Primer (5ʹ to 3ʹ)  ACATGTTCAGCTTTGTGGACC | **IDT** |
| **Mouse** *Timp1* | Forward Primer (5ʹ to 3ʹ)  AGGTGGTCTCGTTGATTTCT  Reverse Primer (5ʹ to 3ʹ)  GTAAGGCCTGTAGCTGTGCC | **IDT** |
| **Mouse** *Il-1β* | Forward Primer (5ʹ to 3ʹ)  GGTCAAAGGTTTGGAAGCAG  Reverse Primer (5ʹ to 3ʹ)  TGTGAAATGCCACCTTTTGA | **IDT** |
| **Mouse** *α-Sma* | Forward Primer (5ʹ to 3ʹ)  GTTCAGTGGTGCCTCTGTCA  Reverse Primer (5ʹ to 3ʹ)  ACTGGGACGACATGGAAAAG | **IDT** |
| **Mouse** *Tnf* | Forward Primer (5ʹ to 3ʹ)  AGGGTCTGGGCCATAGAACT  Reverse Primer (5ʹ to 3ʹ)  CCACCACGCTCTTCTGTCTAC | **IDT** |

- 1. **Biological samples**

| **Description** | **Source** | **Identifier** |
| --- | --- | --- |
| **Human Hepatocytes** |  |  |
| **Human NPCs** |  |  |
| **Human Stellate Cells** |  |  |

- 1. **Deposited data**

| **Name of repository** | **Identifier** | **Link** |
| --- | --- | --- |
|  |  |  |

- 1. **Software**

| **Software name** | **Manufacturer** | **Version** |
| --- | --- | --- |
| **GraphPad Prism** | **GraphPad Software LLC** | **10** |
| **ImageJ** | **NIH; LOCI** | **1.54** |

- 1. **Other (*e.g*. drugs, proteins, vectors etc.)**

| **Name** | **Supplier** | **Cat No#** |
| --- | --- | --- |
| **AY9944** | **Cayman Chemicals** | **366-93-8** |
| **Ethanol Diet** | **Dyets Inc.** | **710362** |
| **High-fat Diet** | **Dyets Inc.** | **710142** |
| **dsiDHCR7** | **IDT** | hs.Ri.DHCR7.13.1 |
| **dsiDHCR7** | **IDT** | hs.Ri.DHCR7.13.2 |
| **dsiDHCR7** | **IDT** | hs.Ri.DHCR7.13.3 |
| **dsiRNA Negative Control** | **IDT** | 51-01-14-03 |

- 1. **Please provide the details of the corresponding methods author for the manuscript:**

| **Tatiana Kisseleva**  **9500 Gilman Drive, #0063, La Jolla, California 92093, USA.**  **Phone: 858.822.5339**  **E-mail: tkisseleva@ucsd.edu** |
| --- |

**2.0 Please confirm for randomised controlled trials all versions of the clinical protocol are included in the submission. These will be published online as supplementary information.**

|  |
| --- |
